# Application of High-Resolution Mass Spectrometry for Ciguatoxin Detection in Fish from the Asia–Pacific Region

**DOI:** 10.3390/toxins17030100

**Published:** 2025-02-20

**Authors:** Xin Li, Ker Lew, Yu Lee Leyau, Ping Shen, Joachim Chua, Kung Ju Lin, Yuansheng Wu, Sheot Harn Chan

**Affiliations:** 1 National Centre for Food Science, Singapore Food Agency, 7 International Business Park, Singapore 609919, Singapore; li_xin@sfa.gov.sg (X.L.); leyau_yu_lee@sfa.gov.sg (Y.L.L.); chan_sheot_harn@sfa.gov.sg (S.H.C.); 2 Department of Food Science & Technology, National University of Singapore, 2 Science Drive, Singapore 117543, Singapore

**Keywords:** HRMS, ciguatoxins, CTX, Indian Ocean ciguatoxins, I-CTX, wild-caught fish in the Asia–Pacific region

## Abstract

Fish is a major source of protein in Asia–Pacific countries. Ciguatera fish poisoning (CFP), caused by consuming reef fish contaminated with ciguatoxins (CTXs), poses a significant health risk, affecting the neurological, gastrointestinal, and cardiovascular systems. Climate change and the global food trade are potentially major factors contributing to the expanding geographical range and frequency of CFP outbreaks. Therefore, the surveillance and monitoring of CTXs in fishery products are essential to safeguard food safety. In this study, liquid chromatography–high-resolution mass spectrometry (LC-HRMS) was used to screen for CTXs in wild-caught fish from the region. Analysis of two grouper fish samples from Okinawa, Japan, detected CTX-1B, a major CTX known to incur in fish from the Asia–Pacific region. Additionally, putative Indian Ocean CTXs (I-CTXs) were also identified. Further study with HRMS on wild-caught red emperor fish from Southeast Asia waters revealed low levels of I-CTXs as well. These findings underscore the urgent need for enhanced food safety measures and expansion of monitoring protocols to include I-CTXs. This research contributes to the global understanding of CTX distribution and confirms the importance of HRMS application in routine surveillance to mitigate the risks associated with ciguatera fish poisoning (CFP).

## 1. Introduction

Ciguatoxin-induced fish poisoning, known as ciguatera, is a significant foodborne illness resulting from the ingestion of certain reef fish contaminated with ciguatoxins (CTXs), which are marine biotoxins produced by the benthic dinoflagellates of the *Gambierdiscus* and *Fukuyoa* genera [1,2,3]. The precursor gambiertoxins and less polar CTXs undergo biotransformation within different fish species into more oxidized and potent versions of CTXs [3,4,5]. This results in the accumulation of CTXs at hazardous levels within the flesh of fish. Exposure to CTXs can lead to symptoms affecting the neurological, gastrointestinal, and cardiovascular systems, depending on the quantity and specific variant of the toxins consumed [6]. In rare, severe instances, such intoxication can result in life-threatening conditions or even death [7,8].

According to the geographic distribution, clinical manifestations, and varied chemical structures, CTXs are identified and characterized into three subfamilies: Pacific CTXs (P-CTXs) [3,9,10,11,12], Caribbean CTXs (C-CTXs) [13,14], and Indian CTXs (I-CTXs) [8,15,16]. P-CTXs are the most studied and diverse group, with over 20 analogs identified [17]. P-CTXs are classified into two subgroups based on their skeletal structures: CTX-1B-type and CTX-3C-type [17]. C-CTXs are found in the Caribbean Sea and the Atlantic Ocean [6]. A total of 12 C-CTXs have been reported [13,18], with the structures of C-CTX-1 and C-CTX-2 determined using NMR and high-performance liquid chromatography coupled with mass spectrometry (HPLC-MS) [13,14]. Later, Fedor et al. elucidated the structures of C-CTX-3 and -4 with the aid of HRMS and chemical derivatization [19]. More recently, C-CTX-5 was identified as a likely precursor toxin of C-CTX-1/2 [20]. In addition to the classical slow-acting ciguatoxins, fast-acting ciguatoxins (FATs) with a molecular weight below 900 Da have also been identified [21]. However, their definitive characterization remains challenging due to limited availability [13,22,23]. For an in-depth review of the chemical and biochemical properties of C-CTXs, including their origin, biotransformation, and the role of analytical chemistry in mitigating public health risks, see Pottier et al. [24]. I-CTXs are found in the Indian Ocean [6]. However, the structures of I-CTXs are not yet fully elucidated [8,15,16].

P-CTX-1 (CTX-1B) is recognized as the most potent activator of voltage-gated sodium channels (VGSC) and is the principal toxin present in carnivorous fish within the Pacific region [6,25]. It accounts for roughly 90% of the toxicity in moray eel [11]. Due to the toxicity, both the Food and Drug Administration of the United States (U.S. FDA) and the European Food Safety Authority (EFSA) have established an extremely low guidance level of 0.01 µg/kg for CTX-1B equivalents in fish [6,26,27]. Owing to the growth of the algae population, the trophic dynamics, and the metabolic alterations within fish, a diverse profile of P-CTX congeners has been observed across various fish species [2,28,29,30].

Despite six I-CTXs being identified since 2002, no other analysis was reported due to the scarcity in ciguatera cases reported in the Indian Ocean (1 case per 10,000 population per annum) [16]. The mortality rate of ciguatera in the Indian Ocean region is significant [8,31,32], and hallucinatory symptoms are commonly reported (3.5% in the cases from 1986 to 1994), albeit they tend to be more anecdotal in nature [33]. There was no guidance level for I-CTXs to date; however, according to Hamilton et al. [15] the potency of I-CTX-1 and -2 is about 60% that of P-CTX-1. From this, Pasinszki et al. considered a safety level of 0.017 µg/kg for I-CTX-1 equivalents [34].

Various methods have been developed for CTX analysis, with the most popular and widely used ones being the mouse bioassay (MBA), biomolecular methods, and chemical methods. The MBA is a traditional method that involves injecting a fish extract into mice and observing them for signs of toxicity [13,18,23,35,36]. It requires no complex analytical equipment. However, it lacks specificity, sensitivity, cannot be automated, and faces ethical concerns [26,34]. Biomolecular methods like cytotoxicity assays, which measure the cytotoxicity of CTXs on cells, typically neuroblastoma (N2a) cells, are simple, sensitive, and automatable but are not cost-effective for routine screening and lack toxin profile information [34,37]. Receptor-binding assays measuring the competition between CTXs and brevetoxins for binding to sodium channel receptor sites are more specific than the MBA but rely on radioactive compounds and receptor sources and cannot be easily automated [38,39,40]. Immunoassays, including the ELISA, are fast and specific but face issues with cross-reactivity and regional specificity, potentially missing CTX-group toxins from other regions [26,41,42,43,44,45,46]. Chemical methods like high-performance liquid chromatography coupled with fluorescence detection (HPLC-FLD) and HPLC coupled with mass spectrometry (HPLC-MS) offer excellent specificity and sensitivity, with HPLC–tandem mass spectrometry (HPLC-MS/MS) being particularly suitable for confirmatory analysis despite being expensive and requiring trained personnel [26,47].

Fish are highly complex matrices, rich in lipids and proteins, making the analysis of CTXs particularly challenging. Although LC-MS/MS using a triple quadrupole system can achieve high sensitivity, the extraction process often results in high levels of co-extractives that negatively impact the analysis [47]. High-resolution mass spectrometry (HRMS) addresses this issue by providing molecular formulas and isotopic patterns of molecules, enabling precise identification of CTX analogs even in complex matrices. This increased selectivity allows for better differentiation of CTXs from matrix interferences and more reliable identification [34].

As a result of the international fish trade, the impact of climate change, and the surge in global tourism, there has been a noticeable uptick in ciguatera cases worldwide [48,49,50,51]. Singapore’s encounter with ciguatera, particularly a case in the year 2000 involving imported fish that led to severe ciguatera poisoning [52], underscores the importance of stringent food safety measures and the need for vigilance in monitoring imported seafood. In our research, HRMS was applied for CTX analysis in two wild-caught groupers from Okinawa, with the identification of not only the expected CTX-1B but also signals corresponding closely to several putative Indian CTXs. Subsequently, analysis was performed on wild-caught fish from Southeast Asia waters, where weak signals of putative I-CTXs were discovered in two red emperors. To the best of our knowledge, this marks the first possible detection of I-CTXs in the region.

## 2. Results

### 2.1. Analysis of CTX1B by HPLC-MS/MS (Unit-Resolution Mass Spectrometry—URMS) and High-Resolution Mass Spectrometry (HRMS)

**Figure 1:**
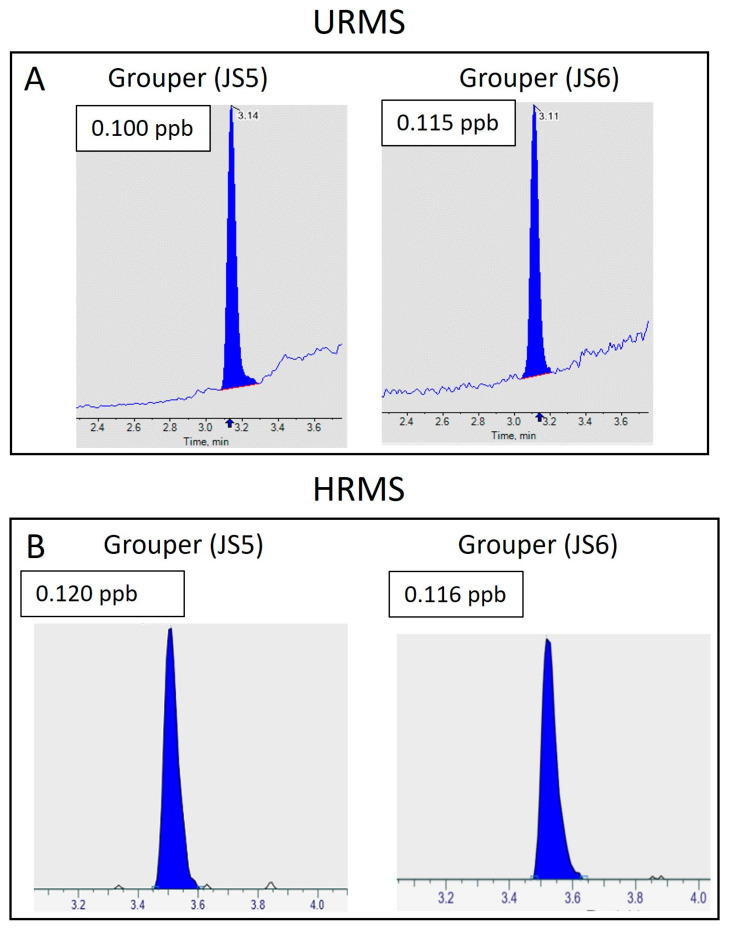
Detection of CTX-1B in grouper JS5 and JS6. (**A**) URMS; (**B**) HRMS.

CTX-1B was detected in two groupers from Okinawa (named as JS5 and JS6) with HPLC-MS/MS and quantified with a matrix-matched calibration curve, covering a linear range from 0.03 µg/kg to 0.1 µg/kg. The concentrations of CTX-1B were 0.100 µg/kg for JS5 and 0.115 µg/kg for JS6, respectively (Figure 1) (calibration curve in Supplementary Figure S1). The grouper samples were subjected to HRMS analysis under parallel reaction monitoring (PRM) mode. Using the same matrix-matched calibration curve, the concentrations of CTX-1B were determined to be 0.120 µg/kg (JS5) and 0.116 µg/kg (JS6) (Figure 1). Both systems detected the incurred CTX-1B with excellent sensitivity. The chromatogram of HRMS showed a much lower baseline as compared to URMS. 

### 2.2. CTX Congener Profiling with High-Resolution Mass Spectrometry (HRMS)

**Figure 2:**
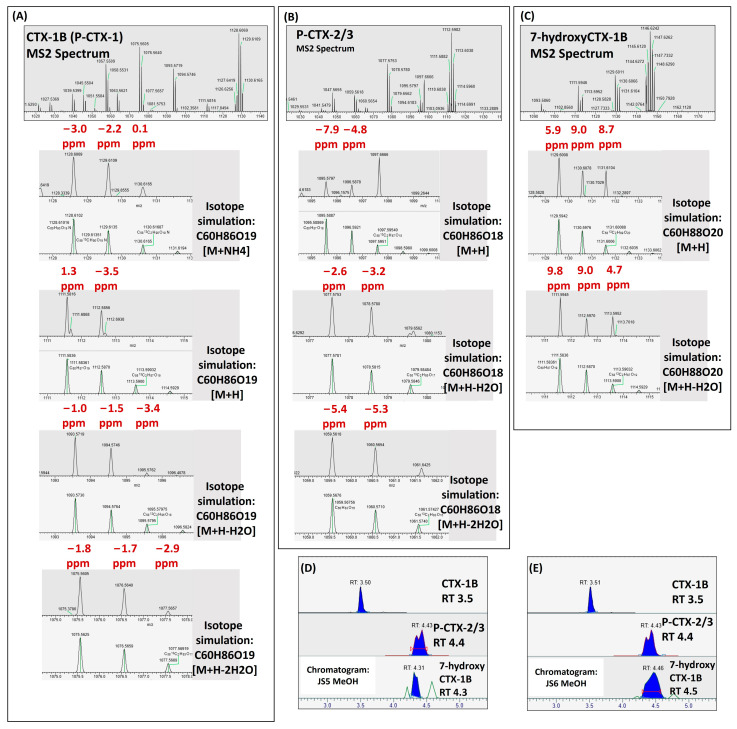
P-CTXs detected in Okinawa groupers. (**A**) MS2 spectrum of CTX-1B; (**B**) MS2 spectrum of P-CTX-2/3; (**C**) MS2 spectrum of 7-hydroxyCTX-1B; (**D**) Chromatogram of P-CTXs in MeOH eluate from sample JS5; (**E**) Chromatogram of P-CTXs in MeOH eluate from sample JS6.

**Figure 3:**
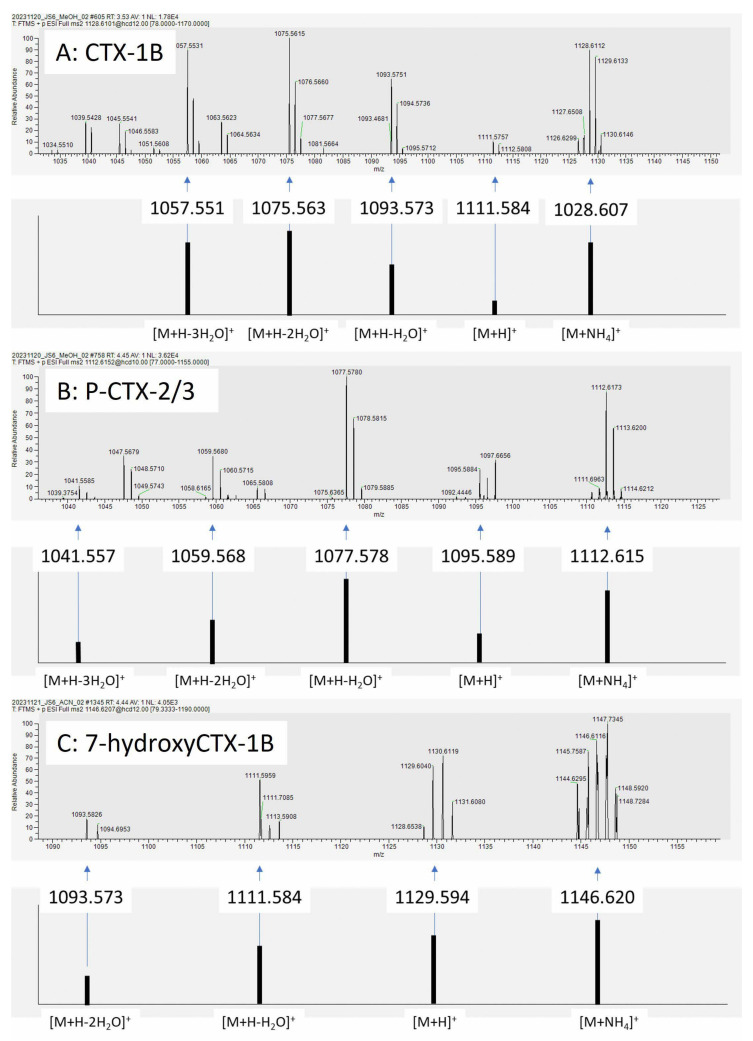
Pseudo spectra of P-CTXs in Okinawa groupers.

We leveraged the unparalleled mass accuracy of HRMS to mitigate the challenge in the lack of commercial CTX standards for most congeners. However, with the strong background the CTX signals were either weak or merged with matrix noise in the MS1 spectra even with HRMS (Supplementary Figure S4). A parallel reaction monitoring (PRM) method, also known as targeted MS2, was utilized instead of a full scan at the MS1 level. Using a targeted PRM method, we monitored 12 Pacific and Indian Ocean CTX congeners (Table 1) by screening the [M+NH4]^+^ precursor ions. By applying a wide isolation window (6 *m*/*z* with 1 *m*/*z* offset), isotopologues were co-fragmented together with the targeted precursors. Therefore, the isotopic patterns, i.e., the isotopic peak mass and abundance, were monitored to provide higher confidence for compound identification. Resolutions of 35,000 or 70,000 were applied to provide fully resolved spectra for the analytes, where the individual peaks corresponding to different molecular species were clearly distinguished from the noisy background. The fragmentation energy (NCE 10 or 12) was optimized based on experiences of the CTX-1B and CTX-3C. Manual interpretation was performed on the MS2 spectra, targeting the fragments from [M+H]^+^ and water-loss peaks, e.g., [M+H-H_2_O]^+^ and [M+H-2H_2_O]^+^. All CTXs were identified with a mass accuracy of 10 ppm for the fragment ions, as well as the matched isotopic peaks of the fragments. In particular, the mass accuracy for all ions in the MS2 spectra was within 10 ppm when compared to the theoretical mass. Moreover, for each fragment at least two isotope ions were detected for all target toxins. The detected P-CTXs are shown in Figure 2. Since there were complex backgrounds, pseudo spectra showing the peak abundance of the possible CTX fragments with the backgrounds removed are shown in Figure 3.

Three P-CTXs were detected, namely CTX-1B, P-CTX 2/3, and 7-hydroxy CTX 1B. The two epimer CTXs P-CTX-2/3 (i.e., 52-epi-54-deoxyCTX-1B and 54-deoxyCTX-1B) [2,3,11,13,53] could not achieve baseline separation in this study. Due to the limited sample quantity, no further optimization of chromatographic conditions was conducted. However, the splitting chromatogram peaks indicates the potential existence of these two epimers. The retention time of CTX-1B and P-CTX-2/3 is 3.5 min and 4.4 min, respectively, indicating a higher hydrophobicity of the latter. The observed CTX profiles are aligned with Yogi’s study [2], where CTX-2/3 were constantly detected together with CTX-1B in all fish samples from Okinawa, but not CTX-3C [2].

### 2.3. Putative I-CTXs Detected in Groupers from Okinawa

**Figure 4:**
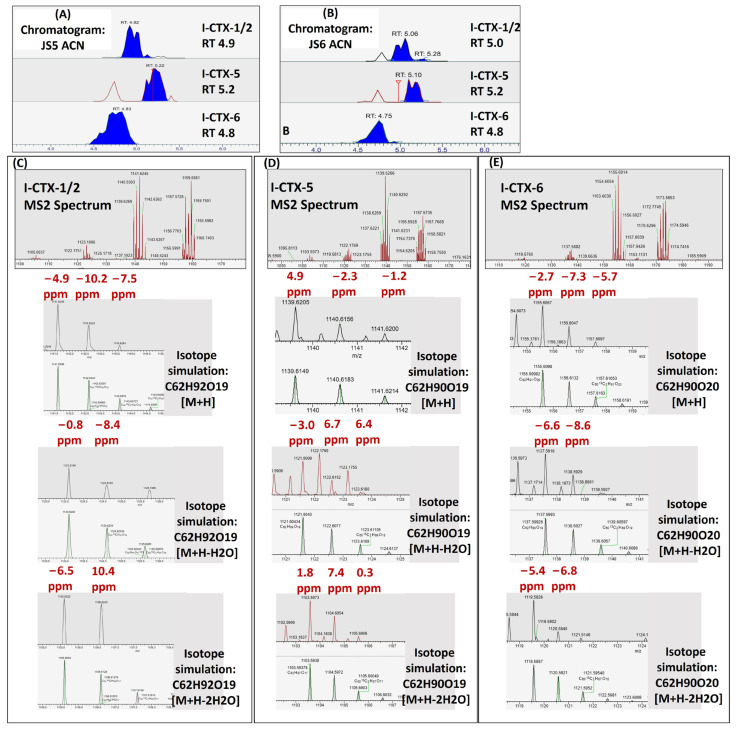
Indian Ocean CTXs detected in Okinawa groupers. (**A**) Chromatogram of I-CTXs in ACN eluate of sample JS5; (**B**) Chromatogram of I-CTXs in ACN eluate of sample JS6; (**C**) MS2 spectrum of I-CTX-1/2; (**D**) MS2 spectrum of I-CTX-5; (**E**) MS2 spectrum of I-CTX-6.

**Figure 5:**
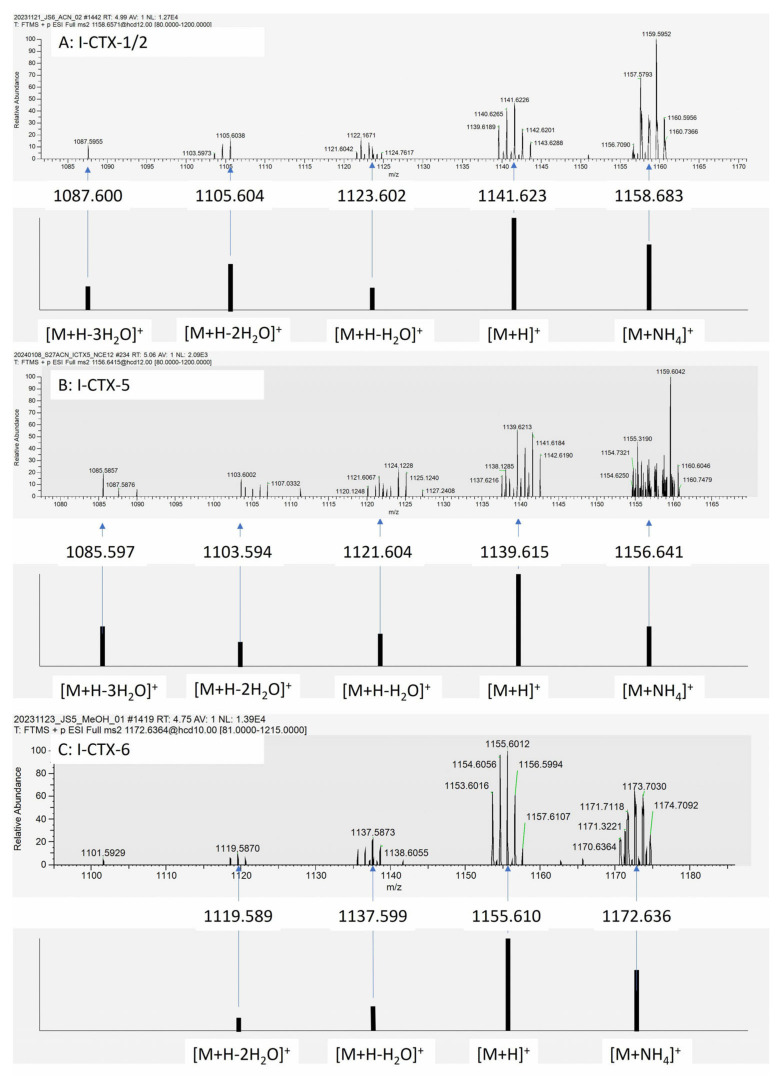
Pseudo spectra of putative I-CTXs in groupers.

Unexpectedly, signals matching with Indian Ocean CTXs I-CTX-1/2, I-CTX-5, and I-CTX-6 were detected in MeOH and ACN eluate from both grouper samples (JS5 and JS6) from Okinawa (Figure 4). The fragments and their isotopic peaks in the MS2 spectra matched with the theoretical mass of [M+H]^+^, [M+H-H_2_O]^+^, and [M+H-2H_2_O]^+^. Since the focus of this study is to monitor a broader range of CTXs with HRMS, no toxin potency bioassays were conducted for the samples. Any detected signals that matched known CTXs were referred to as putative CTXs in this report. The elution order of the putative I-CTX-1/2, I-CTX-5, and I-CTX-6 was in the same order as in the study conducted by Hamilton [8]. I-CTX-1 and I-CTX-2 are isobaric and could only be partially separated. Unlike the reported occurrence in sharks, red bass, and red emperors [8,15,16], I-CTX-3/4 were not detected in the two groupers. The pseudo spectra showing the relative abundance of the fragments of each putative I-CTX are shown in Figure 5.

**Figure 6:**
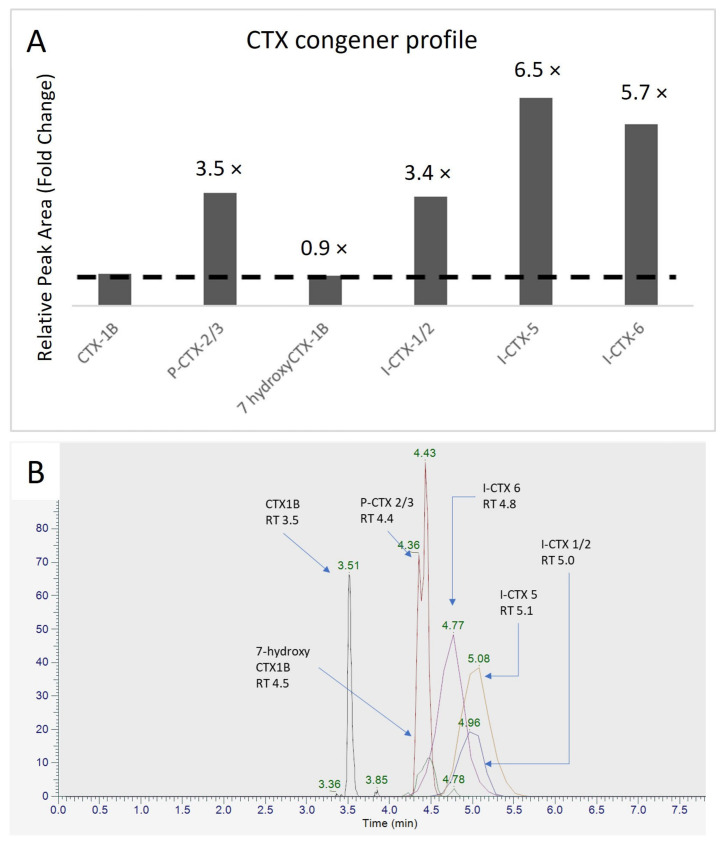
Profile of CTX congeners in Okinawa groupers. (**A**) Relative peak area of the CTX congeners in fold change compared to CTX-1B; (**B**) Chromatogram of the CTX congeners from grouper JS6.

While further investigation is needed to confirm the identity of the putative I-CTXs, an estimation of their potential toxicity has been conducted to underscore the importance of CTX monitoring. With the indistinguishable masses between I-CTX-1 and -2 and C-CTX-1, and given the similar molar responsiveness between P-CTX-1 and C-CTX-1, the assumption was made that there would be a similar molar responsiveness for I-CTXs under the same condition [6,15]. By comparing to the signal intensity of CTX-1B in the same sample (JS6), the signal intensity (sum of the peak area in both MeOH and ACN eluates) was 3.4-fold, 6.5-fold and 5.7-fold of CTX-1B for the putative I-CTX-1/2, I-CTX-5, and I-CTX-6, respectively (Figure 6). The concentration of CTX-1B was 0.116 µg/kg using HRMS quantitation. Based on the assumption made in terms of similar molar responsiveness, the concentration of putative I-CTX-1/2 in JS6 was estimated to be 0.394 µg/kg. This concentration is higher than the safety level of 0.017 µg/kg [34], based on the relative potency for I-CTX-1 and I-CTX-2 being approximately 60% that of CTX-1B [15].

For a larger picture, the total toxicity of CTX-1B equivalents in the sample was estimated based on the following equation:

Total toxicity (µg CTX-1B eq/kg) = ∑(Conc (µg/kg) × TEF)

where:

Conc (µg/kg): Concentration of individual CTX congeners in the sample.

TEF: Toxicity equivalency factor of individual congeners relative to CTX-1B.

TEFs adopted by the EFSA [26] are based on acute i.p. LD50 in mice, where P-CTX-1 = 1, P-CTX-2 = 0.3, P-CTX-3 = 0.3, P-CTX-3C = 0.2, 2,3-dihydroxy PCTX-3C = 0.1, 51-hydroxy P-CTX-3C = 1, P-CTX-4A = 0.1, P-CTX-4B = 0.05, C-CTX-1 = 0.1, and C-CTX-2 = 0.3. For illustration below, TEFs of 0.6 were used for I-CTX-1/2, 5, and 6 [15], with the assumption that TEF = 1 for other CTX congeners with unknown TEFs. 

Taking the example of sample JS6 in Figure 6A, with the assumption of similar molar responsiveness mentioned above, the total toxicity of CTX-1B equivalents in the sample was estimated using the relative peak area of individual congeners:
Total toxicity (µg CTX-1B eq/kg) = {∑(relative peak area × TEF)} × CTX-1B conc           = {1 + (3.5 × 0.3) + (0.9 × 1) + [(3.4 + 6.5 + 5.7) × 0.6]} × 0.116 µg/kg            = 12.31 µg/kg

The total toxicity estimated is much higher than the guidance level of 0.01 µg CTX-1B equivalents/kg fish, indicating a possible gap when only CTX-1B and CTX-3C were analyzed, resulting in 0.116 µg/kg CTX-1B equivalents in the sample. This reinforced the importance of HRMS application as a comprehensive tool to detect CTX congeners for a more complete picture of the CTX occurrence in the sample.

### 2.4. CTX Congener Profiling of Wild-Caught Fish from Singapore Fish Market 

**Figure 7:**
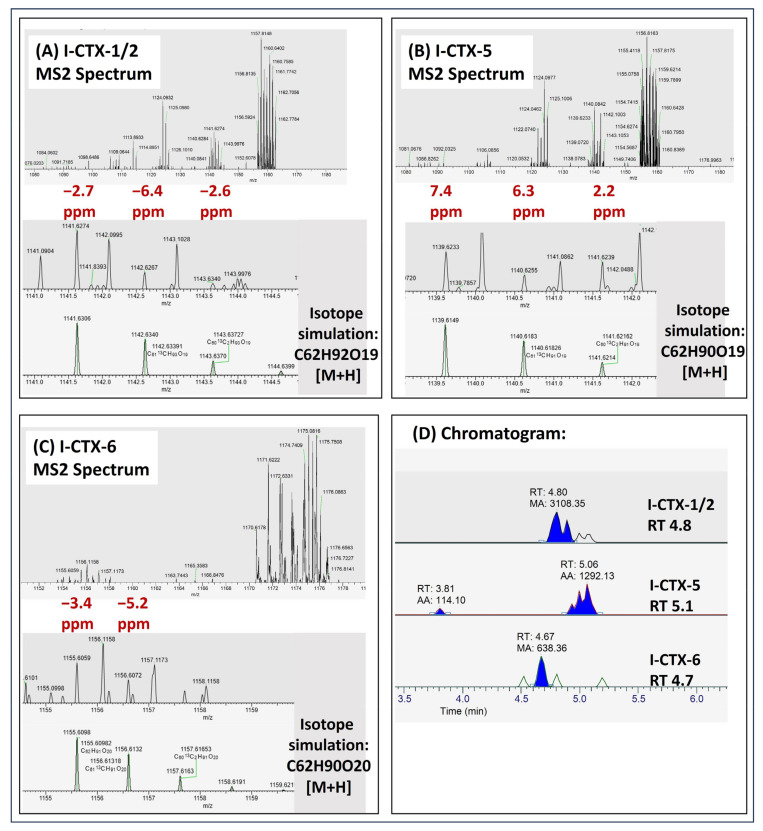
Detection of I-CTXs in red emperors (S13). (**A**) MS2 spectrum of I-CTX-1/2; (**B**) MS2 spectrum of I-CTX-5; (**C**) MS2 spectrum of I-CTX-6; (**D**) Chromatogram of I-CTXs in ACN eluate of sample S13.

**Figure 8:**
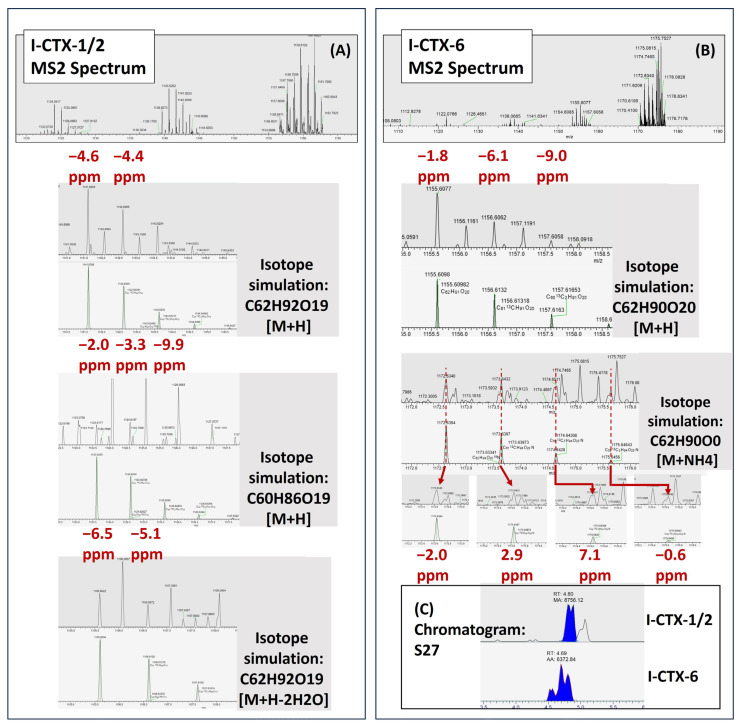
Detection of I-CTXs in red emperors (S27). (**A**) MS2 spectrum of I-CTX-1/2; (**B**) MS2 spectrum of I-CTX-6; (**C**) Chromatogram of I-CTXs in MeOH eluate of sample S27.

As part of baseline monitoring, CTX screening was applied on 13 local wild-caught fishes (Supplementary Data Figures S2 and S3). CTX-1B, CTX-3C, and its intermediates were not detected in any of these wild-caught fish samples. To our surprise, the same putative I-CTX-1/2, I-CTX-5, and I-CTX-6 were detected in two red emperors (Figure 7 and Figure 8). The extracted ion chromatograms shown in the figures were based on the [M+H]^+^ ion.

The two fish were confirmed as *Lutjanus sebae* (red emperors) by the Next-Generation Sequencing (NGS) species identification method. However, precise location information cannot be obtained since it requires further analysis, e.g., whole-genome sequencing. Jurong Fishery Port is licensed by the Singapore Food Agency and serves as the only dedicated fishery port where fishermen from Singapore, Indonesia, and Malaysia unload their daily fresh catch. The red emperors were sourced from Jurong Fishery Port, and we therefore assume that they originated from the neighboring waters of Southeast Asia.

The detection details of putative I-CTXs in red emperors (S13) are shown in Figure 7. Trace amounts of putative I-CTX-1/2, I-CTX-5, and I-CTX-6 were detected. The [M+H]^+^ ion was detected in the PRM MS2 spectrum within 7.5 ppm mass accuracy, with the detection of two-to-three isotopic ions. The retention times of the I-CTXs were similar to our observations in the Okinawa grouper samples.

The PRM method was further optimized to achieve better sensitivity by increasing the ion injection time to 1 Sec, and the target ion count, i.e., automatic gain control (AGC), from 2e^5^ to 5e^5^, and the isolation window from 4 *m*/*z* to 6 *m*/*z*. To better capture the isotopic patterns of CTX analytes, the isolation offset was set to 1, to allow 4 *m*/*z* spectral space at the right side of the targeted mass, instead of 3 *m*/*z* by the default non-offset setting. Two-to-three isotopic clusters can be seen in the MS2 spectra for each fragment. With this method, we detected putative I-CTX-1/2 and I-CTX-6 in another red emperor (sample S27) (Figure 8). For putative I-CTX-1/2, three fragments including [M+H]^+^, [M+H-H_2_O]^+^, and [M+H-2H_2_O]^+^ were detected within 6.5 ppm, with two isotopic variants for these fragments with the same mass accuracy criteria. For the putative I-CTX-6, both the [M+H]^+^ and the precursor [M+NH_4_]^+^ can be seen in the spectrum within 2 ppm. The precursor [M+NH_4_]^+^ of putative I-CTX-6 can be differentiated from the heavy background noise, although some of the isotopic clusters were not baseline-resolved from the interference peaks using the 70,000-resolution scan (Figure 8).

## 3. Discussion

Pacific CTXs (CTX-4A/4B), the CTX precursors from dinoflagellate *Gambierdiscus toxicus* [54], are biotransformed through oxidization to the analogs found in fish, namely CTX-1B (P-CTX-1), 52-epi-54-deoxyCTX-1B (P-CTX-2), and 54-deoxyCTX-1B (P-CTX-3) [4]. These three CTXs co-existed with varying profiles in herbivorous, omnivorous, and carnivorous fishes [29]. CTX-1B is the predominant P-CTX found in predator fishes in the coral reef ecosystem, such as groupers, snappers, wrasses, and moray eels [29]. In this study, we detected CTX-1B and P-CTX-2/3 with comparable chromatogram intensities in Okinawa groupers, although baseline chromatography separation was not achieved for P-CTX-2 and P-CTX-3 due to the limited sample quantity. The elution profile of these toxins matches with previous studies [2,28,30,55]. Notably, CTX-3C was not detected in both grouper samples, consistent with the findings of Yogi et al., which indicated that *G. toxicus* in Okinawa produces only CTX-1B-type toxins [2].

Since Hamilton et al. first identified I-CTX-1, I-CTX-2, I-CTX-3, and I-CTX-4 in 2002, followed by the discovery of I-CTX-5 and I-CTX-6 by Diogene et al. in 2017, no further reports on I-CTXs have been published until now. As a result, information regarding I-CTXs remains limited. In the study conducted by Diogene et al., the elemental compositions of I-CTXs were estimated with a mass tolerance ranging from 4.93 ppm to 6.31 ppm [8]. In our study, the putative I-CTX-1/2 signal was detected with a mass difference ranging from 2.7 ppm to 4.9 ppm compared to the theoretical mass. Similarly, the putative I-CTX-5 signal exhibited a mass difference between 4.9 ppm and 7.4 ppm, while the putative I-CTX-6 signal was observed with a mass difference of 1.8 ppm-to-3.4 ppm. These mass difference ranges are comparable to, or narrower than, those reported in previous studies, underscoring the reliability of our detection.

Currently, only two CTX standards are commercially available, which are CTX-3C and CTX-1B [56]. Although the CTX extraction method successfully reduces the matrix effects to some extent, significant co-eluting interferences persist, contributing to the substantial baseline noise in the chromatogram and complicating the detection of CTXs at low contamination levels. The lack of standards and reference materials remains a crucial hinderance to method development. Particularly for LC-MS detection using unit-resolution systems, targeted methods monitoring analytes within a narrow *m*/*z* window (0.2–2 *m*/*z*) are prone to a high background noise, false positives, or false negatives.

In contrast, HRMS offers a significant advantage for compound identification, especially in complex matrices. Its high mass accuracy and resolution enable precise isotopic pattern recognition, distinguishing target analytes from background noise. This capability is crucial for the detection of CTXs, which a unit-resolution triple quadrupole system could not achieve, particularly in the absence of reference materials or calibrants for I-CTXs (Supplementary Figure S5). Our CTX screening relied on multiple criteria: (1) [M+H]^+^, (2) water-loss peaks, (3) isotopologues, and (4) a chromatogram profile. Despite the limited sample quantities, HRMS provided highly accurate mass measurements that were compared with theoretical masses from the reference studies.

However, the chromatographic peak shapes of P-CTX-2/3, 7-hydroxyCTX-1B, and putative I-CTXs were less sharp as compared to CTX-1B. This discrepancy arises due to the elution of these compounds after 4 min at 95% methanol, resulting in a different retention profile as compared to CTX-1B, which was eluted at 3.5 min, at the point when the gradient had just reached 95% methanol. The effective buffer composition at this stage contained less methanol due to the inherent gradient delay, which is evident in the pump pressure readout. To address these issues, implementing a shallow gradient could help to achieve improved peak shapes for these CTX congeners and enhance chromatographic separation.

**Figure 9:**
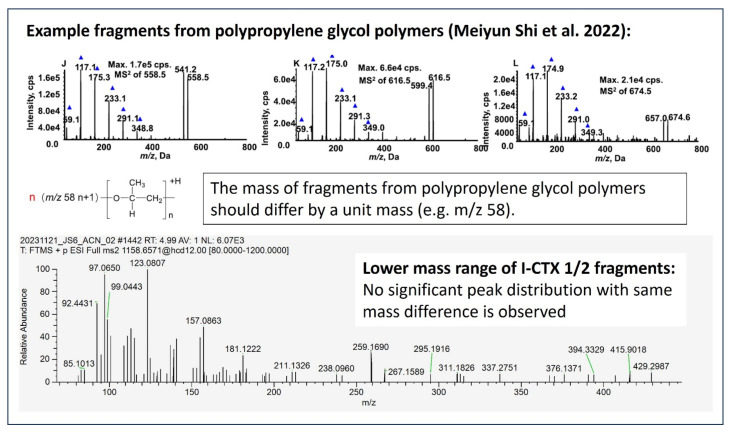
A comparison of putative I-CTX 1/2 fragments to PPG fragments [57].

The possibility of polymer contamination was also considered, given the lack of commercially available standards or validation by bioassay. Studies on polypropylene glycol (PPG) polymers have shown that fragmentation of these polymers results in peaks with consistent mass differences, corresponding to the loss of repeating polymer units [57]. However, in the MS2 spectrum of I-CTX-1/2 from Okinawa groupers, such a peak pattern or any similar polymer fragmentation profile was not observed, ruling out the possibility of the putatively identified I-CTXs as a polymer contamination. A detailed comparison of the I-CTX-1/2 fragments with polypropylene glycol (PPG) fragments is presented in Figure 9.

Although putative I-CTX signals with similar elution profiles have been observed in samples from different regions (Okinawa and Southeast Asia) and various fish species (groupers and red emperors) with accurate mass measurement by HRMS in this study, there remains a risk of false assignment due to the lack of CTX standards and reference materials. Confirmatory techniques, such as mouse neuroblastoma cell assays or radiolabeled ligand-binding assays, are recommended to validate these findings. Future collaborations with laboratories equipped with these assays will enhance the reliability of our results.

Our detection of putative I-CTXs in fish from Okinawa and Southeast Asia provides new evidence on CTX profiles in the Pacific Ocean. This finding warrants attention on the traditional geographical categorization of CTXs, which identified P-CTXs as the predominant causative agents in ciguatera fish poisoning cases in the Pacific Ocean region [58,59,60,61,62,63,64,65,66] and I-CTXs as the primary agents in the Western Indian Ocean (Mauritius and Madagascar) in sharks, red emperors, and red bass, based on limited studies [8,15,16]. It has significant implications for public health and the environment, and presents challenges for epidemiological research and food safety regulation. 

Despite recent detections of putative I-CTXs in the region [44,45,46], our understanding of this toxin group remains limited. Critical knowledge gaps persist regarding their chemical structures, toxicity profiles, and sources. Moreover, the occurrence, distribution, and vectors of I-CTXs in the Indian Ocean remain poorly documented. Given the growing threat of ciguatera fish poisoning, driven by factors such as climate change, the international food trade, and coral reef degradation, there is an urgent need for in-depth research and characterization of I-CTX analogs. This will enable the development of effective risk mitigation strategies, including the publication of codes of practice, consumer advisories, and potentially regulatory limit setting.

The geographical distribution of CTXs typically reflects the regional specificities in the composition of *Gambierdiscus* and *Fukuyoa* species [6,67]. Potentially driven by global warming and climate change, the increasing spread of *Gambierdiscus* and *Fukuyoa* to temperate regions has been reported [68,69]. This spread coincided with ciguatera poisoning outbreaks in NE Atlantic subtropical-temperate regions and in the Mediterranean Sea [70,71,72,73]. Addressing the notable changes in geographical distribution of CTXs, the FAO and WHO’s “Report of the Expert Meeting on Ciguatera Poisoning” now recommends classifying CTXs not only based on geographical distribution, but also on chemical structures [5,50]. The findings of putative I-CTXs in our study provided new evidence on the spreading of CTX-producing dinoflagellates and support for the FAO and WHO’s new classification on CTXs.

## 4. Conclusions

In this study, HRMS has demonstrated its superiority with the accurate mass measurement of precursor and fragment ions, the identification of isotopic clusters, and in comprehensive CTX profiling. HRMS is preferable for the screening of CTX congeners with a high degree of confidence even in the absence of commercially available reference materials. URMS and HRMS complement each other in achieving enhanced sensitivity, specificity, and efficiency in detecting and identifying CTXs at trace levels to ensure the safety of seafood.

With the URMS and HRMS methods, we examined the prevalence and occurrence of CTX-1B in fishery products in Southeast Asia. Putative Indian Ocean CTXs (I-CTX-1/2 5,6) were unexpectedly detected in groupers from Okinawa and wild-caught red emperors from Southeast Asia waters. Certain wild-caught fish, such as giant groupers and red emperors, are commonly used in premium cuisine in Singapore and many other Asian countries. Due to the bioaccumulation of CTX in large carnivorous fish, the risk of ciguatera must not be underestimated in such scenarios. Furthermore, the detection of putative I-CTXs in fish from the Pacific region in this study warrants attention. Given that the potency of I-CTXs was estimated to be as high as 60% of CTX-1B, their unexpected presence in the Pacific region’s fishery products could pose a significant food safety risk. Therefore, regulatory bodies in the region should consider expanding their monitoring scope to include I-CTXs, as well as the implementation of HRMS for marine biotoxin screening to enhance surveillance capacity. 

Despite the currently low impact of CTX detection in Singapore, and since marine wild-caught fish still constitute a significant part of fishery products in Asia [74], it is essential to increase public awareness of ciguatera through education, industry and media engagement, and community outreach to empower consumers to make informed choices about seafood consumption, thereby protecting their health and well-being.

## 5. Materials and Methods

### 5.1. Sample Collection

#### 5.1.1. Incurred Fish Samples

Two groupers (JS5–JS6) caught in Okinawa Japan in 2019 were detected with CTX-1B at a level of 0.08 µg/kg by JFRL.

#### 5.1.2. Wild-Caught Fish from Southeast Asia Waters 

Thirteen wild-caught fish including mackerel, red snappers, red emperors, white snappers, giant groupers, and stingrays, were obtained from a family-owned shop at a local fish market between September 2023 and January 2024. These fish, sourced from Jurong Fishery Port, were purchased at intervals ranging from every few days to once a week. These wild-caught fishes were claimed to be caught in Southeast Asia waters.

### 5.2. Fish Species Identification by Next-Generation Sequencing (NGS)

The total genomic DNA from fish samples was extracted using the DNeasy^®^ Blood & Tissue Kit (Qiagen, Hilden, Germany) according to the manufacturer’s protocol. The concentrations of isolated DNA and the DNA library pool were determined by using the Qubit^®^ 4.0 fluorometer and the Qubit Broad Range (BR) Assay Kit following the manufacturer’s instructions (Thermo Fisher Scientific, Waltham, MA, USA).

The Ion Torrent™ Next-Generation Sequencing platform incorporating the SGS™ All Species ID Fish DNA Analyser Kit (Thermo Fisher Scientific, Waltham, MA, USA) was employed in this study for fish species identification.

### 5.3. Chemicals and Reagents

CTX-1B (100 ng, product number 038-25801, lot ACQ1948) was purchased from FUJIFILM Wako Pure Chemical Corporation (Tokyo, Japan) and reconstituted in 1 mL methanol. Solutions were stored in glass vials at −20 °C.

The diethyl ether used was of AR grade. The acetone, n-hexane, methanol, and acetonitrile used were of HPLC grade or LC-MS grade. Acetone and n-hexane were from Tedia (Fairfield, CT, USA), diethyl ether was from Merck (Darmstadt, Germany), methanol (MeOH) was from EAM (Selangor, Malaysia), and Acetonitrile (ACN) was from Anhui Fulltime Specialized Solvents and Reagents Co., Ltd. (Anqing, China). Ammonium formate (AR grade) was acquired from Acros Organics (Geel, Belgium) and formic acid (AR grade) was from Thermo Fisher Scientific (Waltham, MA, USA). Ultra-pure water was obtained from the ELGA PureLab Ultra Analytic ultra-pure polishing system (Veolia, High Wycombe, UK), with a resistivity of 18.2 MΩ-cm.

### 5.4. Sample Preparation

Flesh was carefully cut out from the fish and blended using a food processor into a fine paste.

#### Sample Extraction

The CTX extraction method for LC-MS/MS analysis was adapted from previously published protocols by Lewis et al. [75] and Yogi et al. [2,76]. The homogenized flesh sample portion of 5 g was first extracted with acetone (15 mL, twice) followed by centrifugation. The combined supernatant was condensed to a syrup under a nitrogen stream set at 50 °C. Organic contents were then extracted from the syrup with diethyl ether (5 mL, twice), after which the combined organic layers were completely dried under nitrogen stream at 50 °C. The residue was suspended with 90% methanol (*v*/*v*, 1.5 mL) and defatted with hexane (3 mL, twice). This was followed by drying the methanolic solution under a nitrogen stream at 50 °C. The crude extract was dissolved with an ethyl acetate/methanol mixture (9:1, *v*/*v*, 5 mL) and passed through a Florisil cartridge (500 mg, GL Sciences Inc., Tokyo, Japan). Next, the eluate was completely dried under a nitrogen stream at 50 °C. The resulting residue was dissolved using acetonitrile (5 mL) and applied to a primary and secondary amine cartridge (PSA, 200 mg, GL Sciences Inc., Tokyo, Japan). The low polar analogs were eluted with acetonitrile (5 mL, ACN eluate), followed by the elution of hydroxylated analogs with methanol (3 mL, MeOH eluate). Both ACN and MeOH eluates were dried separately and reconstituted in 10mM ammonium formate in 70% methanol (0.25 mL), filtered through a 0.1 µm PTFE filter, and submitted to LC-MS/MS analysis. The injection volume of 10 µL, equivalent to 0.2 g of fish flesh, was applied for instrument analysis.

### 5.5. Instrumentation

The analyses were conducted using two systems:

System A: Ultra-High-Performance Liquid Chromatography (UHPLC) coupled with Unit-Resolution Triple Quadrupole (QqQ) Mass Spectrometry;

System B: Ultra-High-Performance Liquid Chromatography (UHPLC) coupled with a benchtop Quadrupole-Orbitrap High-Resolution Mass Spectrometer.

#### 5.5.1. UHPLC Conditions

A linear gradient using 5 mM ammonium formate in water with 0.1% formic acid as eluent A and 100% methanol (MeOH) as eluent B with an ACUITY UPLC BEH C18 column (2.1 mm × 100 mm) was set up for the chromatographic separation of CTX. The flow rate was 0.35 mL min^−1^, the injection volume was 10 μL, and the column temperature 40 °C. The elution gradient was as follows: 70% B to 95% B from 0 to 3 min, hold at 95% B for 3 min, decrease from 95% to 70% B in 0.1 min, and hold for 2 min at 70% B.

#### 5.5.2. System A with a Unit-Resolution MS Detector

System A is composed of a UHPLC system (Agilent Technologies, Waldbronn, Germany) coupled with a triple quadrupole 6500^+^ QTRAP mass spectrometer equipped with a TurboV^®^ electrospray ionization source (ESI) (SCIEX, Framingham, MA, USA). Mass spectrometric detection was performed in positive ionization mode using multiple reaction monitoring (MRM) mode. The optimized ESI^+^ parameters were as follows: curtain gas at 30 psi, ion spray at 5500 V, turbo gas temperature at 300 °C, Gas 1 and 2 at 50 and 70 psi, respectively, and an entrance potential at 10 V. The MRM acquisition method for CTX-1B was created using the scheduled MRM algorithm. The MRM method scans two transitions corresponding to 1128.6/1075.6 ([M+NH_4_]^+^/[M+H-2H_2_O]^+^) and 1128.6/1057.6 ([M+NH_4_]^+^/[M+H-3H_2_O]^+^). The dwell times and cycle time are optimized to provide a better peak detection and improve reproducibility. A detection window of 90 sec and a target scan time of 0.35 sec were chosen for the analysis. The instrument control and data processing were conducted using Analyst software 1.6.3 (SCIEX, Framingham, MA, USA) and MultiQuant 3.0.2.

#### 5.5.3. System B with a High-Resolution MS Detector

System B is composed of a Dionex Ultimate 3000 UHPLC coupled with a benchtop Quadrupole-Orbitrap high-resolution mass spectrometer (Thermo Fisher Scientific, Bremen, Germany). The instrument was operated in parallel reaction monitoring (PRM) mode to achieve the best sensitivity. Acquisition was carried out in positive ionization mode, with a capillary temperature at 275 °C, a sheath gas at 35 (arbitrary unit), and an auxiliary gas at 12.5 (arbitrary unit). The probe heater temperature was set at 300 °C. The spray voltage was set at 3800 V. The S-Lens RF level was set at 60. Mass spectra were acquired by MS/MS at the resolution of 35,000 or 70,000, with a 6 *m*/*z* isolation window (1.0 *m*/*z* offset to include more isotopes of the targeted ions). The AGC was set to 2e^5^ with 300 ms maximum injection time (max IT), or 5e^5^ with 1000 ms max IT. The Normalized Collision Energy (NCE) was set at 10 or 12 (arbitrary unit). The spectra were recorded in profile mode. The PRM targets the [M+NH_4_]^+^ ion of each CTX and generates fragment ions in the MS/MS spectrum.

#### 5.5.4. Matrix-Matched Calibration

Matrix-matched calibration standards of CTX-1B were prepared, with concentrations ranging from 0.03 µg/kg to 0.1 µg/kg in a 5 g blank fish extract. The blank fish sample was analyzed and determined to be free of CTXs.

A quality control standard of 1 µg/kg CTX-1B in 10 mM ammonium formate in 70% methanol was prepared freshly for each batch of the analysis to ensure the system was in the prime condition before sample injection. 

##### Limit of Detection and Limit of Quantitation (System A)

The limit of detection (LOD) and the limit of quantification (LOQ) for CTX-1B were determined to be 0.01 µg/kg (S/N > 3) and 0.03 µg/kg (S/N > 10), respectively, through 12 replicate spikes in blank fish samples in seven occasions. The amounts of 5 µL and 15 µL of 10 µg/kg CTX-1B standard were spiked into 5 g of blank fish samples and proceeded through the same protocol for sample extraction.

The maximum %CV of the LOD and LOQ was 15.65% and 15.15%, respectively. It was noticed that different types of fish yielded different background noises in the LC-MS/MS. Spiked samples of blank fish matrices were carried out on groupers, red snappers, and seabream (Supplementary Figure S6).

## Figures and Tables

**Table 1:** CTX congeners monitored with HRMS.

**CTX4A group**	**Name**	**Formula**
	CTX-1B	C_60_H_86_O_19_
	CTX-4A/B	C_60_H_84_O_16_
	M-seco-CTX-4A/B	C_60_H_86_O_17_
	52-epi-54-deoxyCTX-1B 54-deoxyCTX-1B (P-CTX-2/3)	C_60_H_86_O_18_
**CTX3C group**	**Name**	**Formula**
	CTX-3C	C_57_H_82_O_16_
	2,3,51-trihydroxyCTX-3C	C_57_H_84_O_1_9
**I-CTX group**	**Name**	**Formula**
	I-CTX-1/2	C_62_H_92_O_19_
	I-CTX-3/4	C_62_H_92_O_20_

## Data Availability

The original contributions presented in this study are included in the article/Supplementary Materials. Further inquiries can be directed to the corresponding author.

## Supplementary Materials

The following supporting information can be downloaded at https://www.mdpi.com/article/10.3390/toxins17030100/s1, Figure S1: Matrix-matched calibration curve of CTX-1B; Figures S2, S3: Chromatogram of HRMS screening performed on local fish samples; Figure S4: Spectrum background of CTX-1B and P-CTX-2/3 on the MS1 level; Figure S5: Chromatogram of I-CTXs with different mass tolerances; Figure S6: LOD and LOQ in spiked samples; Figure S7: The original chromatogram of putative I-CTX-1/2 in local red emperors (S13) and in Okinawa samples; Figure S8: Okinawa-incurred fish samples JS5 and JS6, with known CTX-1B concentrations, were used as quality controls (QCs) for comparison with the putative I-CTX-1/2 signal; Figure S9: Local wild-caught red emperors (S13) with matrix-matched CTX-1B as the QC; Figure S10: Local wild-caught red emperors (S27) with matrix-matched CTX-1B as the QC; Table S1: Mass of detected CTXs in Okinawa and Singapore local fish samples; Table S2: Summary of the CTX-1B recovery rate in pre-spiked red snappers; Table S3: Method validation—Validation parameters and method performance.

## Abbreviations

The following abbreviations are used in this manuscript:

CFP	ciguatera fish poisoning
P-CTX	Pacific ciguatoxins
I-CTX	Indian Ocean ciguatoxins
C-CTX	Caribbean ciguatoxins
MBA	mouse bioassay
HPLC-MS	HPLC coupled with mass spectrometry
HRMS	high-resolution mass spectrometry
URMS	unit-resolution mass spectrometry
PRM	parallel reaction monitoring
MRM	multiple reaction monitoring
AGC	automatic gain control
PPG	polypropylene glycol
LOD	limit of detection
LOQ	limit of quantification
