# Supplementary material for: Application of High-Resolution Mass Spectrometry for Ciguatoxin Detection in Fish from the Asia–Pacific Region
Source: Toxins (Basel). 2025 Feb 20;17(3):100. doi: 10.3390/toxins17030100 (PMC11946577; doi:10.3390/toxins17030100)
Supplement: Supplementary file 1 [file toxins-17-00100-s001.zip › toxins-3413042-supplementary.pdf]

Supplementary Materials: Application of High-Resolution Mass Spectrometry for Ciguatoxin Detection in Fish from the Asia–Pacific Region

Xin Li, Ker Lew, Yu Lee Leyau, Ping Shen, Joachim Chua, Kung Ju Lin, Yuansheng Wu and Sheot Harn Chan

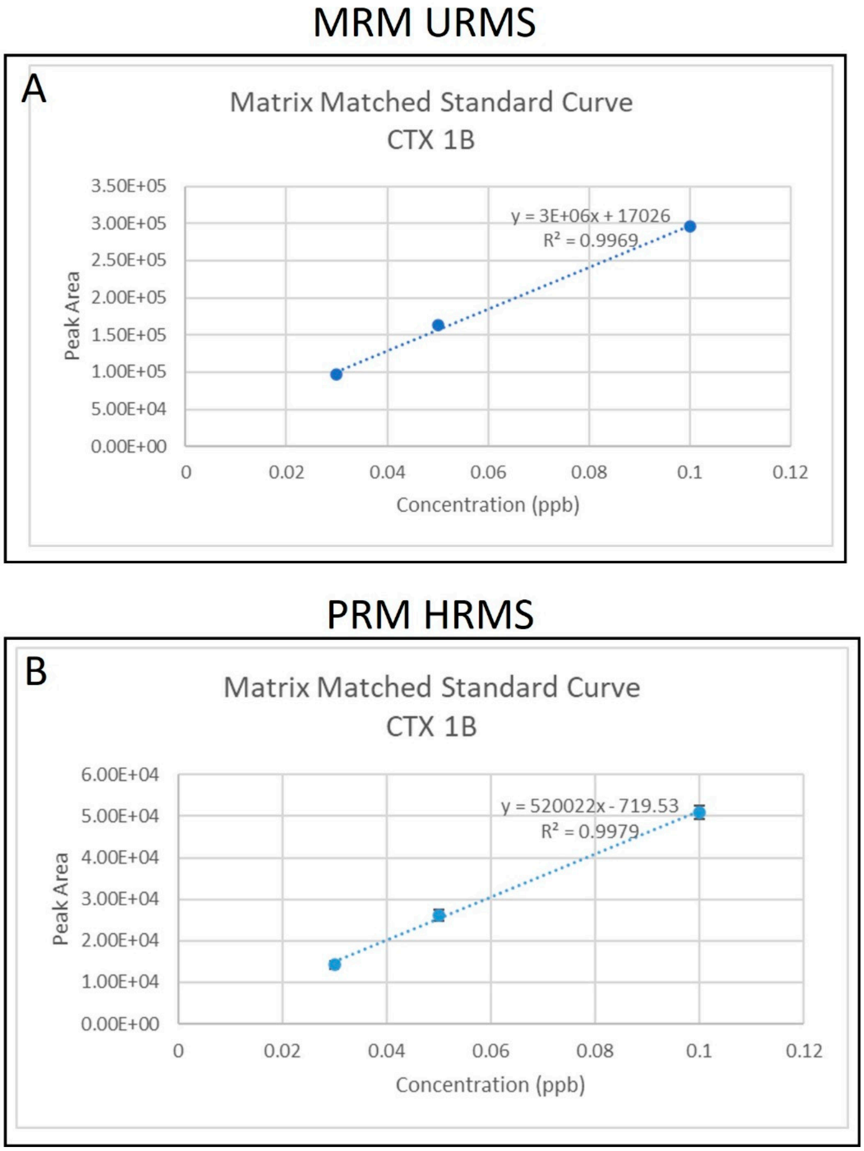

Figure S1. (**A**) Matrix matched calibration curve of CTX-1B measured by triple-quadrupole; (**B**) Matrix matched calibration curve of CTX-1B measured by HRMS.

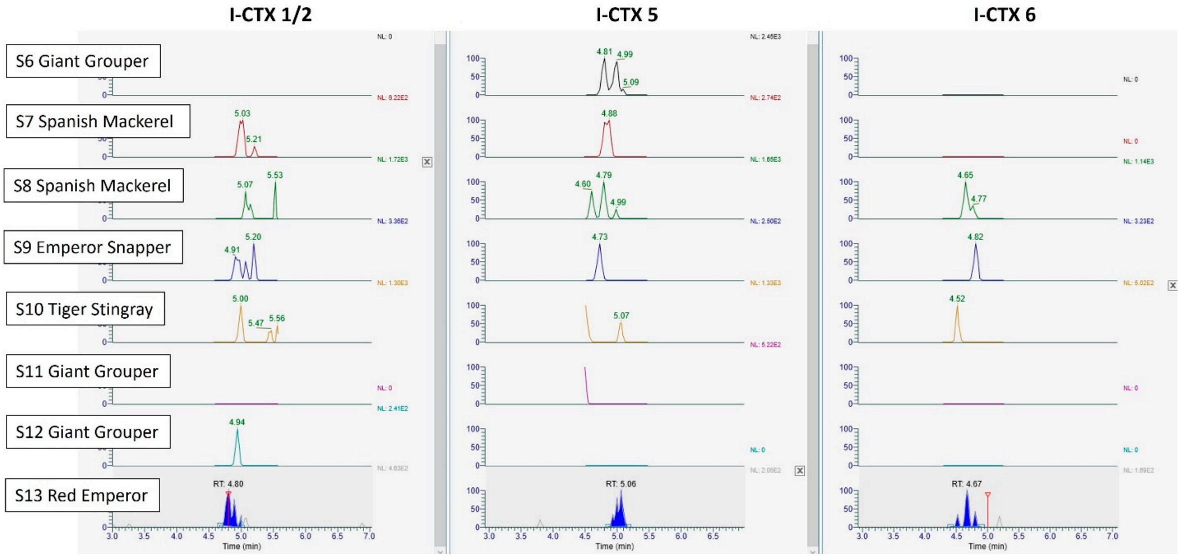

Figure S2. Chromatogram of HRMS screening performed on local fish samples. The chromatogram is based on $[M+H]^+$. Only detections in S13 and S27, which met the criteria of mass accuracy, isotopologues and water-loss peaks, were considered in current study.

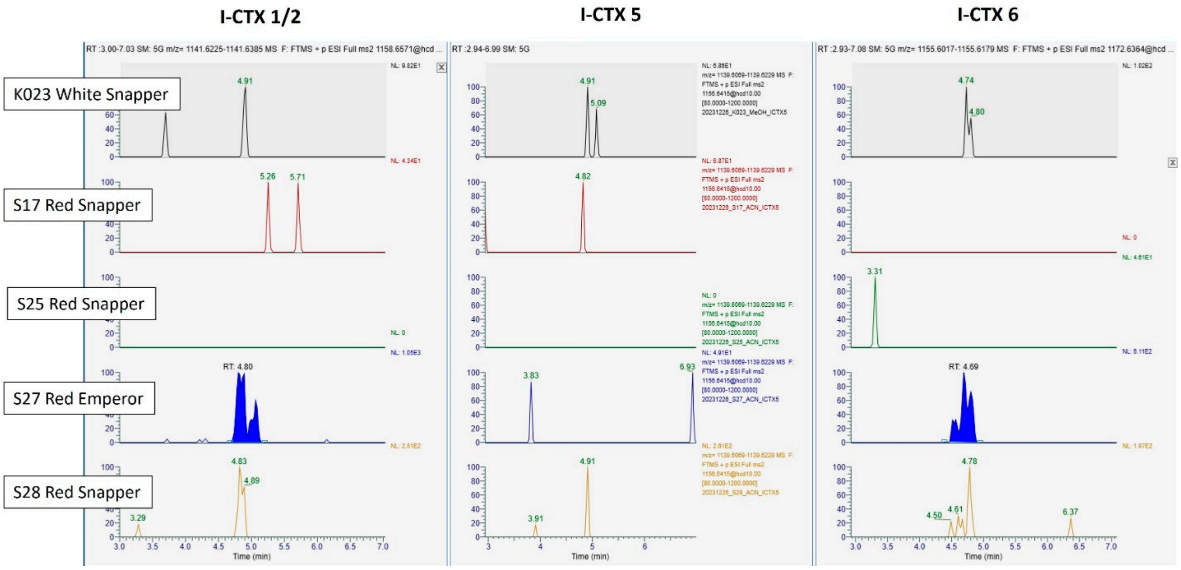

Figure S3. Chromatogram of HRMS screening performed on local fish samples. The chromatogram is based on $[M+H]^+$. Only detections in S13 and S27, which met the criteria of mass accuracy, isotopologues and water-loss peaks, were considered in current study.

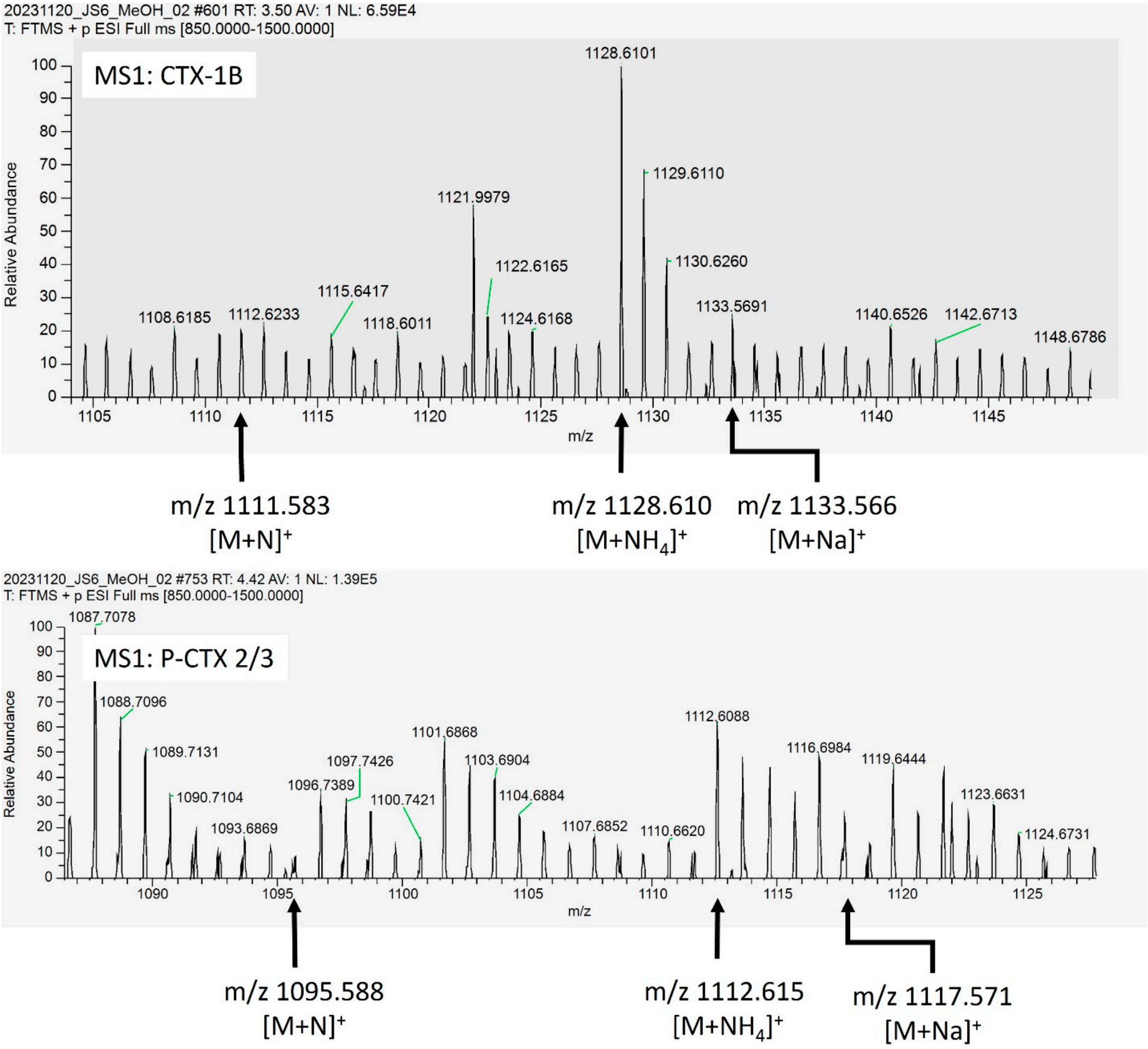

Figure S4. Strong background at the MS1 level was captured using HRMS. The CTX-1B and P-CTX 2/3, especially the latter, were almost indistinguishable from the chemical noise.

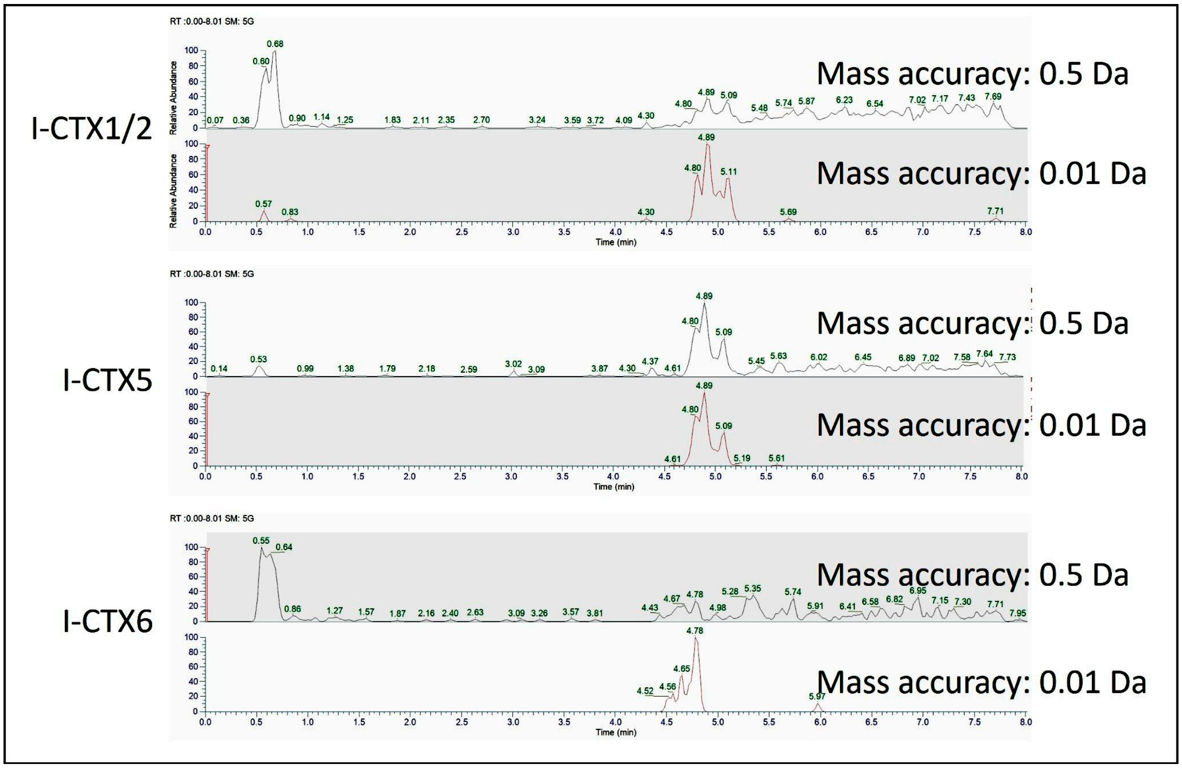

Figure S5. Different mass tolerance, 0.5 Da and 0.01 Da, were used to generate the extracted ion chromatography (EIC). With 0.5 Da, any mass within ± 0.5 Da of the target mass was considered as the target, and its intensity was used for plotting the chromatogram. With 0.01 Da, only mass within ± 0.01 Da of the target mass was considered. Hence, less noise was included in the EIC using 0.01 Da, and the baseline of the chromatography was lower. This clearly shows that higher mass accuracy and mass resolution facilitate the detection of I-CTXs.

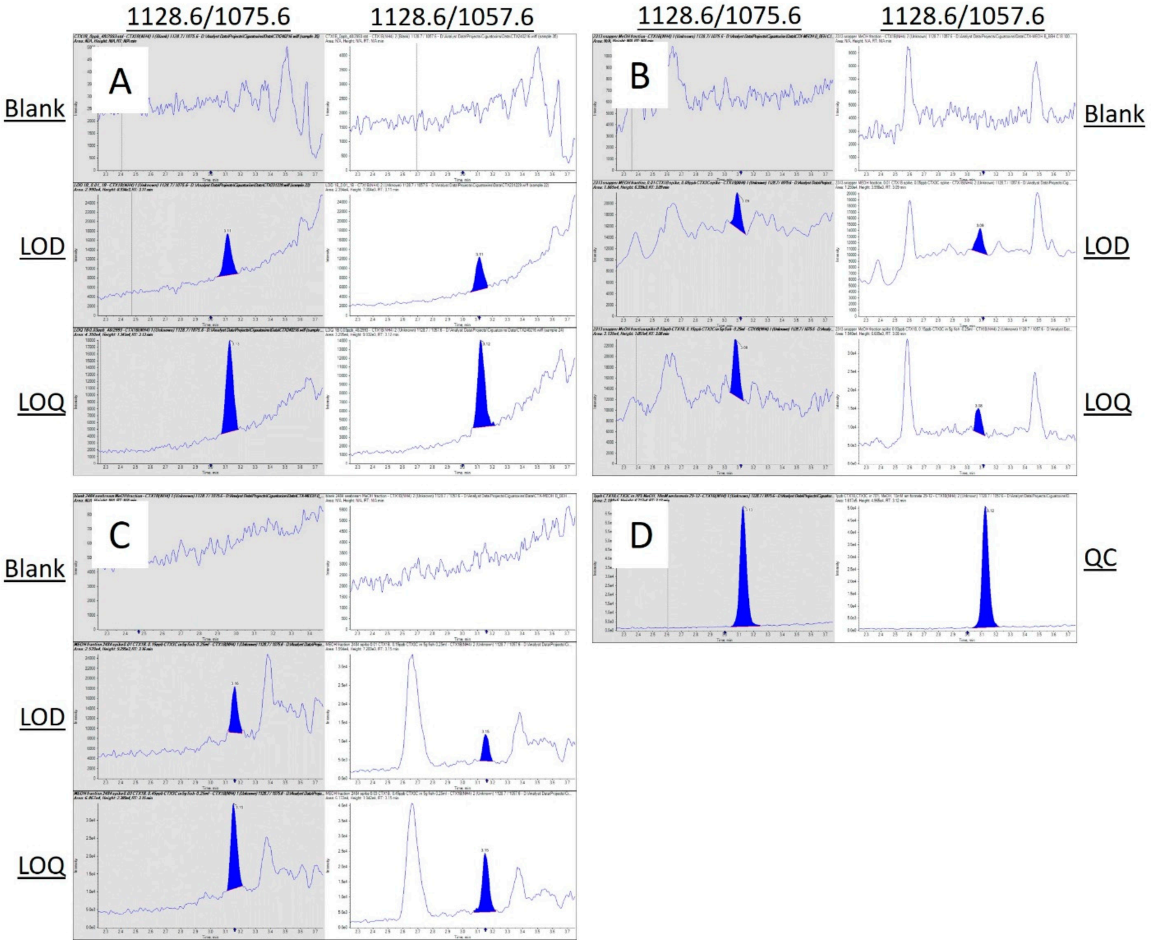

Figure S6. The limit of detection (LOD) and the limit of quantification (LOQ) for CTX-1B on triple quadrupole system. (**A**) grouper (**B**) red snapper (**C**) seabream (**D**) standard in 10mM ammonium formate in 70% methanol.

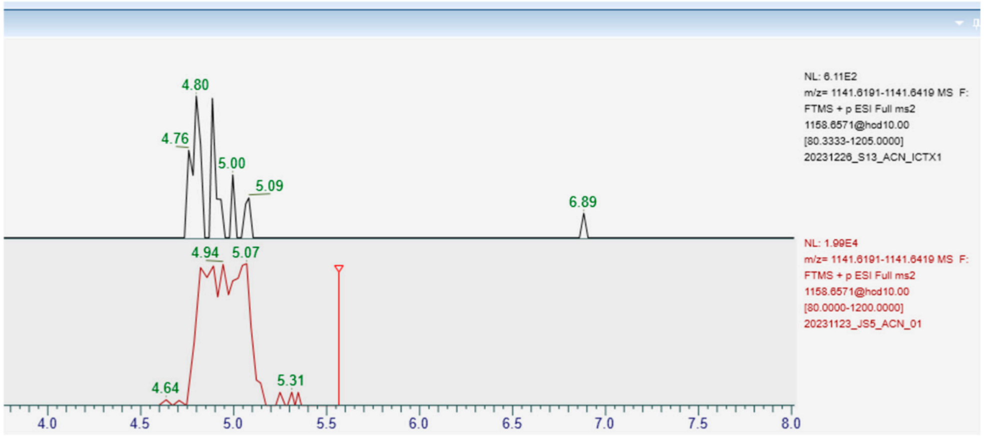

Figure S7. The original chromatogram (top) of putative I-CTX-1/2 in local red emperor (S13) and in Okinawa samples (bottom), which are both without smoothing. The chromatogram of S13 has splitting peaks due to unstable spray and interference.

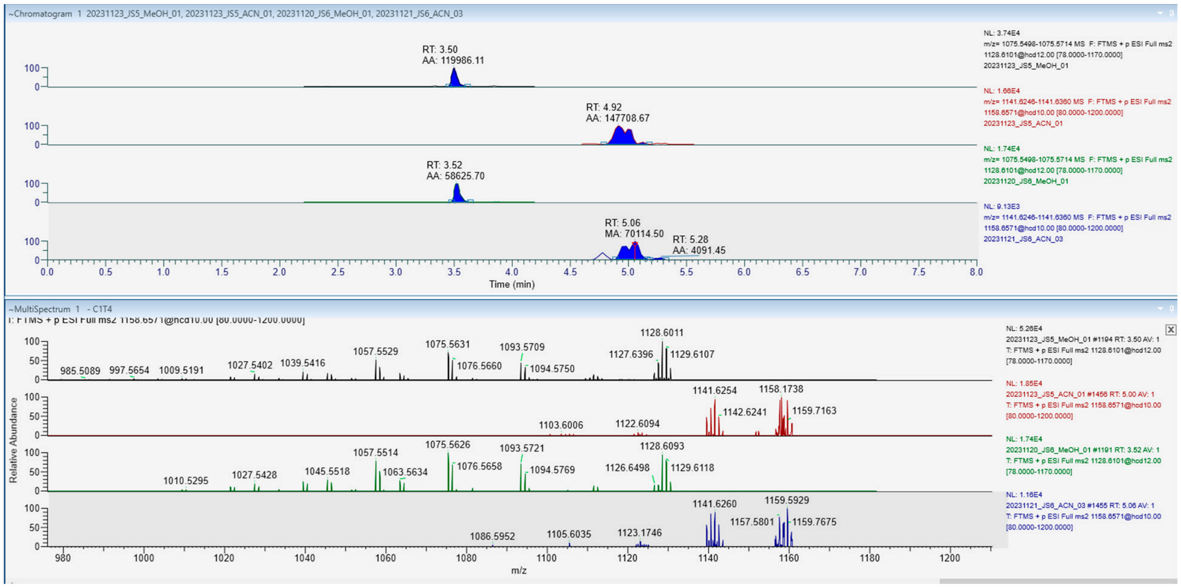

Figure S8. Okinawa incurred fish JS5 and JS6 with known CTX-1B concentration served as QC (chromatogram/spectrum: JS5 at 1st row, JS6 at 3rd row). The putative I-CTX signal were detected in the same samples (chromatogram/spectrum: JS5 at 2nd row, JS6 at 4th row).

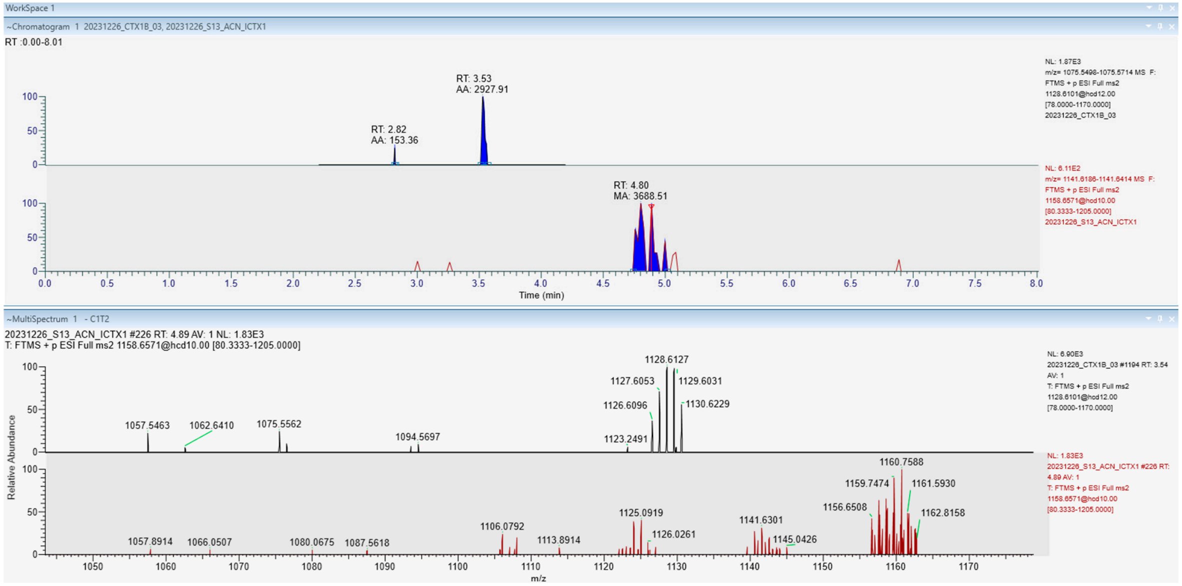

Figure S9. Local wild-caught sample S13(chromatogram/spectrum in 2nd row) with matrix-matched CTX-1B (chromatogram/spectrum in 1st row) as QC.

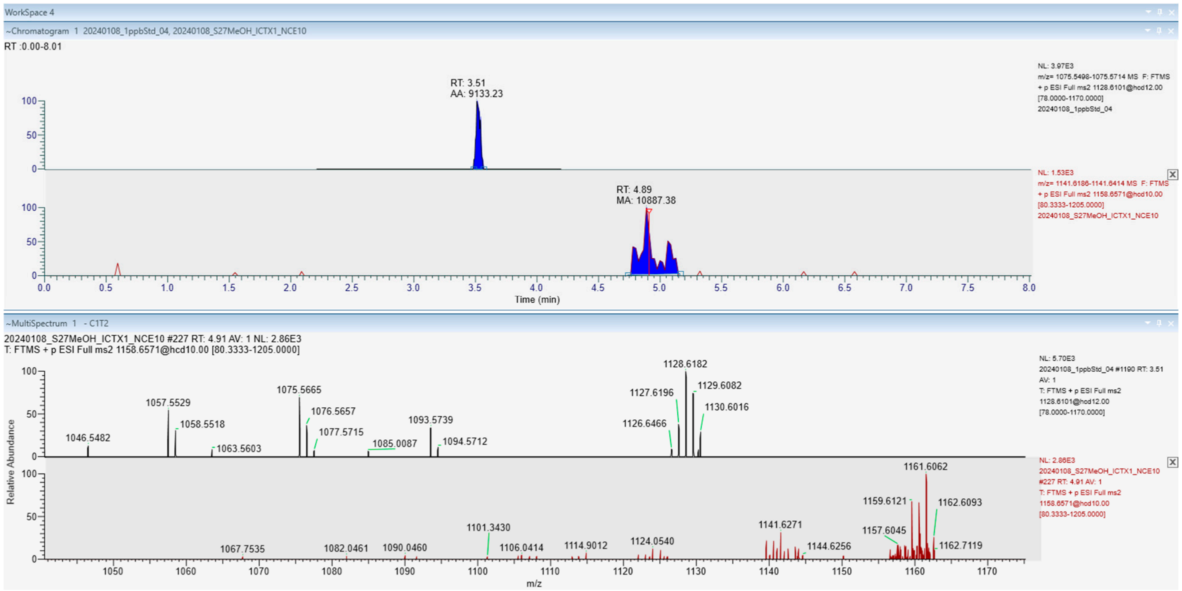

Figure S10. Local wild-caught sample S27(chromatogram/spectrum in 2nd row) with matrix-matched CTX-1B (chromatogram/spectrum in 1st row) as QC.

Table S1. Mass of detected CTXs in Okinawa and local fish samples.

	**Formula**	**Theoretical mass**	**Okinawa grouper**	**Red emperor (S13)**	**Red emperor (S27)**
CTX-1B	C60H86O19	1128.6102	1128.6069		
		[M+NH4]	-3.0 ppm		
P-CTX-2/3	C60H86O18	1095.5887	1095.5797		
		[M+H]	-7.9 ppm		
7-hydroxyCTX-1B	C60H88O20	1129.5942	1129.6008		
		[M+H]	5.9 ppm		
I-CTX-1/2	C62H92O19	1141.6306	1141.6249	1141.6274	1141.6253
		[M+H]	-4.9 ppm	-4.9 ppm	-4.6 ppm
I-CTX-5	C62H90O19	1139.6149	1139.6205	1139.6233	
		[M+H]	4.9 ppm	7.4 ppm	
I-CTX-6	C62H90O20	1155.6098	1155.6067	1155.6059	1155.6077
		[M+H]	-2.7 ppm	-3.4 ppm	-1.8 ppm

Table S2. Summary of CTX-1B recovery rate in pre-spiked red snapper.

	Spike level	
	0.01 μg/kg	0.03 μg/kg
Occasion 1	88.7	88.6
	71.2	84.4
	71.9	63.9
Occassion2	82.6	73.8
	55.8	69.9
	77.7	80.2
Occassion3	86.5	71.4
	91.9	67.6
	79.0	68.7
Ave	78.4	74.3
std dev	11.05	8.33
%CV	14.10	11.21

Table S3. Method validation—Validation parameters and method performance.

S/N	Validation Parameters	Performance	Remarks
1	Linearity	$R^2 \geq 0.99$<br>Linear range 0.03 – 0.1 μg/kg	3-point Matrix-matched calibration curve over 2 occasions at 0.03, 0.05 and 1 μg/kg
2	Accuracy	62.5 – 75.0%	Using Okinawa incurred samples with known CTX-1B concentration
3	Repeatability (Precision)	< 20% RSD *	2 concentration levels over 7 occasions
4	Reproducibility (Intermediate Precision)	< 45% RSD *	2 concentration levels over 7 occasions
5	Recovery	50 – 120% *	2 concentration levels over 3 occasions
6	Limit of detection (LOD)	0.01 μg/kg	7 independent data
7	Limit of Quantitation (LOD)	0.03 μg/kg	7 independent data

* in accordance with AOAC guidelines for Standard Method Performance Requirements for analyte of 1 μg/kg.
